# Are young female suicides increasing? A comparison of sex-specific rates and characteristics of youth suicides in Australia over 2004–2014

**DOI:** 10.1186/s12889-019-7742-9

**Published:** 2019-10-28

**Authors:** Nina Stefanac, Sarah Hetrick, Carol Hulbert, Matthew J. Spittal, Katrina Witt, Jo Robinson

**Affiliations:** 1 The University of Melbourne, Parkville, Australia; 2 Orygen, The National Centre of Excellence in Youth Mental Health, 35 Poplar Road, Parkville, VIC 3052 Australia; 3 The University of Auckland, Auckland, New Zealand; 4 Turning Point, Eastern Health Clinical School, Monash University, Richmond, Australia

**Keywords:** Suicide, Youth, Young people, Females, Australia, Risk factors, Epidemiology

## Abstract

**Background:**

Australian mortality statistics suggest that young female suicides have increased since 2004 in comparison to young males, a pattern documented across other Western high-income countries. This may indicate a need for more targeted and multifaceted youth suicide prevention efforts. However, sex-based time trends are yet to be tested empirically within a comprehensive Australian sample. The aim of this study was to examine changes over time in sex-based rates and characteristics of all suicides among young people in Australia (2004–2014).

**Methods:**

National Coronial Information System and Australian Bureau of Statistics data provided annual suicide counts and rates for 10–24-year-olds in Australia (2004–2014), stratified by sex, age group, Indigenous status and methods. Negative binomial regressions estimated time trends in population-stratified rates, and multinomial logistic regressions estimated time trends by major suicide methods (i.e., hanging, drug poisoning).

**Results:**

Between 2004 and 2014, 3709 young Australians aged 10–24 years died by suicide. Whilst, overall, youth suicide rates did not increase significantly in Australia between 2004 and 2014, there was a significant increase in suicide rates for females (incident rate ratio [IRR] 1.03, 95% confidence interval [CI] 1.01 to 1.06), but not males. Rates were consistently higher among Aboriginal/Torres Strait Islander youth, males, and in older (20–24-years) as compared to younger (15–19 years) age groups. Overall, the odds of using hanging as a method of suicide increased over time among both males and females, whilst the odds of using drug-poisoning did not change over this period.

**Conclusions:**

We showed that suicide rates among young females, but not young males, increased over the study period. Patterns were observed in the use of major suicide methods with hanging the most frequently used method among both sexes and more likely among younger and Aboriginal/Torres Strait Islander groups. Findings highlight the need to broaden current conceptualizations of youth suicide to one increasingly involving young females, and strengthen the case for a multifaceted prevention approach that capitalize on young females’ greater help-seeking propensity.

## Background

Suicide is the leading cause of death among young females in Australia (aged 15–24 years), and the second leading cause among 15–19 year-old females worldwide [1–3]. Recent Australian mortality data indicate that the suicide rate for young females has increased steadily over the past 10 years while the rate for young males has fluctuated, despite remaining comparatively higher [3]. Increases in young female suicide rates have been observed across other Organization for Economic Co-operation and Development (OECD) countries, including New Zealand, the United States (US), Canada, Sweden, the United Kingdom (UK), and Finland [4–9]. In addition, a comparative study of OECD countries showed sharp decreases in suicide rates among male adolescents across several European countries, while female rates remained stable or increased [9]. These time trends across the OECD suggest that rates of suicide are changing among young females and raise the possibility that public health approaches to prevention have not adequately targeted the factors contributing to this increase. Consistent with this, an epidemiological study of 21 OECD nations showed that government-led programs were associated with declines in suicide rates among males but not females, and among young people specifically, declines were greater among males than females [10]. Furthermore, Australian research has shown most local prevention programs have had negligible effects on national rates [11], and no impact on suicide rates in young women [12, 13].

Given observed increases in young female suicide across the OECD and the significant public health burden conferred by suicide, an examination of time trends in sex-specific rates and characteristics of youth suicides in Australia is timely. This could inform the development of targeted and effective prevention strategies in order to attenuate suicide rates. Recent population studies have highlighted several time trends in epidemiological factors known to be associated with increased suicide risk among young people, which may have contributed to rising rates among young females in Australia. These include a growing number of early adolescent (10–14-year-old) females at risk [6, 14], elevated rates among young Aboriginal and Torres Strait Islander (Indigenous) females [3, 15–18], and growing use of lethal methods (specifically hanging) [6, 19].

### Age and development

Consistent with data reporting conventions, we define young people as those aged between 10 and 24 years, and further categorize this group to include early adolescents (10–14 years), late adolescents (15–19 years) and young adults (20–24 years) [20]. The transition from adolescence to young adulthood marks a period of rapid change [21–23]. In particular, high prevalence mental disorders, which are significantly associated with heightened risk of suicide, typically emerge during this period, and continue into young adulthood [24]. Indeed, depression and anxiety, which are common, show greater continuity into early adulthood among females compared with males [25]. As such, existing research supports the transition to adolescence as marking the beginning of a period of heightened suicide risk in young females.

Several OECD studies have documented recent growth in suicide rates among females as young as 10-years-old, and show rates increase across the adolescent years. Recent population studies in Finland, the US and Canada found rates have increased significantly in female adolescents and young adults over the past two decades. Canadian and US data further indicate that the greatest increase occurred among 10–14-year-old females, while overall rates remained significantly higher among 15–24-year-old females [6, 14].

### Indigenous status

Indigenous young people are consistently over-represented in Australian suicide statistics, raising the possibility that a rise in young female suicide rates may be driven in part by increases among young Aboriginal and Torres Strait Islander females in particular. National statistics indicate rates are four times higher among 15–24-year-old Indigenous females and three times higher among Indigenous males compared with their non-Indigenous peers [15]. Furthermore, between 2007-11 and 2012–16, Australian statistics showed crude mortality rates increased among young Indigenous females alongside declining rates among same-aged males [3, 18]. Increases among Indigenous females have also been documented for specific regions; over 1994–2007 in Queensland [16], and over 2005–14 in the Kimberley region of Western Australia [17]. Risk of suicide within Indigenous communities is underpinned by a myriad of systemic factors contributing to significant disadvantage in health and socio-economic outcomes (for a review see: Zubrick and colleagues [26]). Population studies have also shown that rates of depression and anxiety among Indigenous youth have increased [27]. Hence, a growing proportion of young Indigenous females may be experiencing poor social and emotional wellbeing which, in turn, may be contributing to increased suicide rates.

### Suicide methods

A further factor that may be contributing to possible increases in young female suicides relates to changes in suicide methods used by young females. Method lethality refers to methods which increase the probability of a fatal outcome [28], and is a well-documented risk factor for youth suicide. Typically, males have used more lethal methods of suicide (e.g. hanging and firearms [29]) as compared to females. Accordingly, males account for more completed suicides [15] while females account for more non-fatal attempts [1, 30]. The relationship between specific methods and the magnitude of suicide rates was illustrated by a New Zealand study which showed that sex differences in completed and attempted youth suicides were completely explained by males’ use of more lethal methods [31]. Thus, if young females have increased their use of more lethal methods this may be contributing to a rise in suicide rates. Indeed, research has shown a rise in the use of lethal methods of self-harm and suicide among young females [6, 14, 19, 32, 33], and hanging is now the leading method among female youth in both Australia and across the OECD [6, 7, 15, 19, 34, 35].

Whilst these factors have been investigated for their impact on youth suicide individually, the contribution of these factors in concert has never been examined in a nationwide cohort of young people to date. The present study therefore aimed to examine sex-specific suicide rates among 10–24-year-olds in Australia over the period 2004 to 2014, with a key focus on investigating whether or not key epidemiological risk factors (i.e., age, and Indigenous background) may explain changes in female suicide rates over time. Our secondary aim was therefore to examine if one explanation for the change in rates was increasing use of hanging (a highly lethal method).

## Method

### Design

This descriptive study employed a retrospective case series design, including all suicides and probable suicides in individuals aged 10–24-years in Australia, between 2004 and 2014.

### Data sources

#### Suicide data

The National Coronial Information System (NCIS) is an online data repository of all deaths reported to Australian coroners [36]. The NCIS reports demographic information, external causes and mechanisms of death as recorded in coronial files. The quality and completeness of this information varies between cases due to legislative differences across jurisdictions, and is often delayed due to coronial processes [37]. Therefore, deaths occurring within the period 2004–2014 were selected for inclusion in order to balance data recency with completeness.

We extracted data from NCIS on all closed cases where the deceased was aged 10–24-years, died between 1st January 2004 and 31st December 2014 and where the intent was categorized as ‘intentional self-harm’ or ‘undetermined’.

We extracted data on year of death, sex, age at death (in years), Indigenous origin, residential state/territory, International Statistical Classification of Diseases and Related Health Problems, 10th Revision (ICD-10) code, medical cause of death, primary and secondary mechanisms of injury.

Several adjustments were made to prepare the data for analysis. Indigenous origin data were collapsed into a binary variable representing two groups: ‘Aboriginal/Torres Strait Islander origin’ vs ‘non-Indigenous origin or unknown’. This enabled us to compare rates between groups with well-documented disparities in suicide risk. Age at death was coded into three categories: ‘10–14-year-old’, ‘15–19-year-old’ and ‘20–24-year-old’. Suicide methods were classified by ICD-10 codes cross-referenced with cause of death and mechanism data, to reflect methods used rather than medical cause of death [38]. Three categories of methods were examined: hanging (X70), drug poisoning (X60 to X65), and all other methods (including 11 cases using unknown methods).

#### Population data

Mid-year estimated resident populations were obtained from the Australian Bureau of Statistics, stratified by year, sex, age group, and Indigenous status [39]. We used this data to calculate annual crude mortality rates (CMR) per 100,000 person-years and as an offset term for regression analyses to estimate rates over time.

### Statistical analysis

Statistical analyses, including multinomial logistic regressions and negative binomial regressions, were conducted in Stata, version 15.1.

#### Time trend analyses

We estimated trends in suicide rates over time stratified by age and Indigenous origin using negative binomial regression. This method is similar to Poisson regression but adjusts for possible over-dispersion in the data (occurring when the variance exceeds the mean). Our outcome was the number of deaths for groups defined by the age, sex, Indigenous origin and year group covariate pattern. Our models included an offset term to account for population size, and predictors for sex, age group, and Indigenous status, and interaction terms between these predictors and year of death.

An initial model examined the total sample, and subsequent models examined each sex. In each instance, our first model included all predictors, while subsequent models removed non-significant interaction terms. We present the results of our final, best fitting models.

#### Methods of suicide

We used individual-level data to examine predictors of major suicide methods (i.e., hanging, drug poisoning) as compared to all other methods using multinomial logistic regressions. Predictors were as above.

Counts and proportions by socio-demographic characteristics described the sample, crude rates were calculated by sex, age-group, indigenous status and year.

## Results

Between 2004 and 2014, 3709 Australians aged 10–24 years died by suicide and 75% were male. Regarding missing data, more than 10% of cases had Indigenous status recorded ‘unlikely to be known’ (14.7%), as did 0.3% of cases for suicide methods, reflecting difficulties ascertaining this information from coronial data.

The CMR was 11.2 per 100,000 person-years (PY) for males and 4.1 per 100,000 PY for females (Table 1). The rates increased by age group for both sexes, and Indigenous males and females were both at elevated risk of suicide (30.1 and 13.8 per 100,000 PY, respectively). Rates were highest in the Northern Territory and Western Australia and lowest in Victoria, New South Wales and South Australia (Table 1).

**Table 1:** Suicide counts and crude mortality rates (per 100,000 person-years) by sex, 2004–2014

	Females		Males		Total	
	Count	Rate	Count	Rate	Count	Rate
Total	961	4.11	2748	11.17	3709	7.72
Age						
10–14yo	57	.76	82	1.05	139	.91
15–19yo	394	5.12	924	11.40	1318	8.3
20–24yo	510	6.15	1742	20.09	2252	13.27
Aboriginal/TSI	149	13.79	347	30.76	496	22.46
No/unknown	812	3.64	2401	10.22	3213	7.01
State/Territory of Residence						
NSW	216	2.89	629	8.01	845	5.51
VIC	218	3.77	602	9.91	820	6.92
QLD	249	5.19	676	13.53	925	9.44
SA	71	4.35	218	12.27	289	8.33
WA	134	5.43	382	14.56	516	10.14
TAS	24	4.55	66	11.84	90	8.30
NT	38	14.31	123	41.73	161	28.73
ACT	6	1.43	45	10.24	51	5.94
N/A^a^	4	–	6	–	10	–
Year of Death						
2004	84	4.16	254	12.04	338	8.19
2005	69	3.38	261	12.21	330	7.89
2006	73	3.54	253	11.71	326	7.72
2007	78	3.73	255	11.61	333	7.77
2008	69	3.25	241	10.77	310	7.11
2009	80	3.72	231	10.15	311	7.02
2010	88	4.06	218	9.55	306	6.88
2011	105	4.84	260	11.39	365	8.19
2012	121	5.54	251	10.95	372	8.31
2013	95	4.33	272	11.78	367	8.15
2014	99	4.49	252	10.83	351	7.74

*Aboriginal/TSI* Aboriginal and/or Torres Strait Islander people, *NSW* New South Wales, *VIC* Victoria, *QLD* Queensland, *SA* South Australia, *WA* Western Australia, *TAS* Tasmania, *NT* Northern Territory, *ACT* Australian Capital Territory

^a^Not applicable (includes overseas residents or those with no fixed address)

**Fig. 1:**
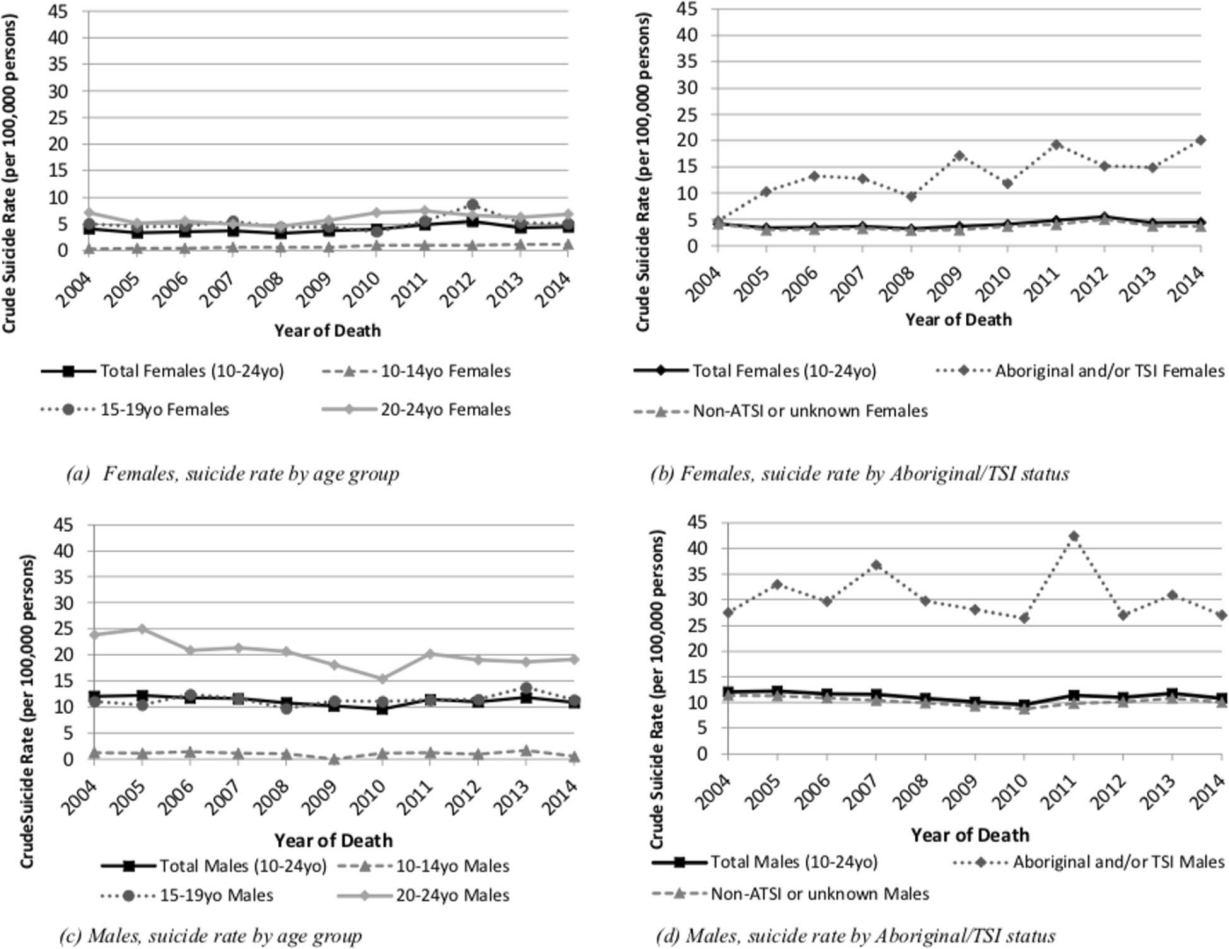
Plots of CMR (per 100,000 person-years) by sex, age group and Indigenous (Aboriginal/TSI) status, 2004–2014. **a** Females, suicide rate by age group. **b** Females, suicide rate by Aboriginal/TSI status. **c** Males, suicide rate by age group. **d** Males, suicide rate by Aboriginal/TSI status

Figure 1 presents a series of population-stratified graphs, plotting time trends in annual CMR. For both sexes, rates among older and Indigenous young people were consistently higher than younger and non-Indigenous young people, respectively. Among males, rates for 20–24-year-olds exhibited a distinct downward trend (Fig. 1c), while among females, clear upward trends were observed for 10–14-year-olds (Fig. 1a) and Indigenous females (Fig. 1b). Comparing Fig. 1a with Fig. 1c, rates for 10–14-year-old females exceeded their male peers in 2009 (0.59 vs. 0), 2012 (1.03 vs. 0.98) and 2014 (1.17 vs. 0.55).

### Time trend analyses

Negative binomial regression revealed rates for young males were significantly higher compared to rates among young females (Incident Rate Ratio [IRR] = 2.56, 95% CI: 2.30–2.85). There was no statistically significant change over time in the overall suicide rate (IRR = 1.01, 95% CI: 0.98–1.04), but when stratified by sex, the rate for females rose by 3% per year over the period (IRR = 1.03, 95% CI: 1.01–1.06). Using this model parameter, and assuming a linear relationship between suicide prevalence and time, this equates to a total increase of 38% (1.03^11) (see Table 2). Table 2 also shows the rate of suicide among Indigenous females was four times higher than for other females (IRR = 4.12, 95% CI: 3.41–4.98). Additionally, female rates differed significantly by age; compared with the 10–14-year-old age group, suicide rates were 6.80 (95% CI: 5.07–9.10) times higher among the 15–19-year-old age group, and 8.33 (95% CI: 6.24–11.11) times higher among the 20–24 age group (Table 2).

**Table 2:** Best-fitting negative binomial regression model for each sex, examining suicide rates over time, by age and Indigenous groups

Predictors	IRR	95% CIs	*p*	*Pseudo R* ^*2*^
Females				
Time	1.03	1.01–1.06	.014	0.32
Age group^a^			< .0001	
15–19 years	6.80	5.07–9.10		
20–24 years	8.33	6.24–11.11		
Aboriginal/TSI^b^	4.12	3.41–4.98	< .0001	
Males				
Time	0.98	0.91–1.05	.556	0.34
Age group^a^			< .0001	
15–19 years	9.59	6.34–14.52		
20–24 years	20.89	13.94–31.30		
Age group x Time^a^			.018	
15–19 years	1.03	0.96–1.11		
20–24 years	0.99	0.92–1.06		
Aboriginal/TSI^b^	3.42	3.05–3.83	< .0001	

*IRR* Incident rate ratio, *95% CIs* 95% Confidence Intervals, *Aboriginal/TSI* Aboriginal and/or Torres Strait Islander origin

^a^‘10–14-year-old’ age group was the reference category

^b^‘Non−/Unknown Aboriginal or Torres Strait Islander' origin group was the reference category

The picture was more complex for males, as shown in Table 2. Among the 10–14-year-old age group (IRR = 0.98, 95% CI: 0.91–1.05) and the 15–19-year-old age group (IRR = 0.98*1.03 = 1.01, 95% CI: 0.99 to 1.03) there was no evidence of change over time. For the 20–24-year-old age group, the rate decreased by 3% per year (IRR = 0.98*0.99 = 0.97, 95% CI: 0.96 to 0.99). Once again, assuming a linear relationship between suicide prevalence and time, this model parameter equates to a 28% decline overall for this group. The suicide rate was more than three times higher among Aboriginal/Torres Strait Islander males than for other males (IRR = 3.42, 95% CI: 3.05–3.83).

### Suicide methods

Most young people used hanging (67.1% females, 66.5% males); whereas a smaller proportion used drug-poisoning (9.4% females, 3.2% males), or other methods (23.5% females, 30.3% males). Table 3 presents the results of separate multinomial logistic regression models for youth and each sex by suicide method.

**Table 3:** Final multinomial logistic regression models for suicide methods over time, by sex, age and Indigenous groups

	Hanging			Poisoning		
	OR	95% CIs	*p*	OR	95% CIs	*p*
All Youth						
Time	1.03	1.01–1.06	.003	1.00	0.96–1.05	.906
Sex^a^	1.06	0.90–1.25	.481	0.29	0.21–0.39	<.001
Age group^b^			<.001			.003
15–19 years	0.79	0.51–1.23	.306	1.76	0.54–5.79	.352
20–24 years	0.61	0.39–0.93	.023	3.00	0.93–9.68	.065
Aboriginal/TSI^c^	9.67	6.55–14.26	<.001	0.18	0.72–0.43	<.001
Females						
Time	1.00	0.96–1.04	.936	1.01	0.94–1.08	.836
Age group^b^			.009			.022
15–19 years	0.58	0.28–1.20	.143	1.85	0.42–8.04	.414
20–24 years	0.41	0.20–0.85	.017	3.30	0.78–13.97	.105
Aboriginal/TSI^b^	6.54	3.56–12.03	<.001	0.25	0.09–0.69	.008
Males						
Time	1.05	1.02–1.07	.001	0.99	0.93–1.07	.955
Age group^b^			.021			.12
15–19 years	0.96	0.55–1.66	.878	1.57	0.21–11.91	.661
20–24 years	0.75	0.44–1.29	.302	2.54	0.35–18.64	.359
Aboriginal/TSI^c^	12.14	7.30–20.21	<.001	0.08	0.01–0.59	.013

*OR* Odds ratio, *CIs* 95% Confidence Intervals, *Aboriginal/TSI* Aboriginal and/or Torres Strait Islander

^a^‘Females’ served as the reference category

^b^‘10–14-year-old’ age-group served as the reference category

^c^‘Non−/Unknown Aboriginal/Torres Strait Islander status’ group served as the reference category

#### Hanging

Overall, the odds of using hanging as a method of suicide increased over time among youth (Odds Ratio [OR] = 1.03, 95% CI: 1.01–1.06); females and males showed no difference in their odds of using hanging (*p* = .48). Compared to 10–14-year-olds, 20–24-year-old youth showed lower odds of using hanging (OR = 0.61, 95% CI: 0.39–0.93); and Indigenous youth had over 9 times greater odds of dying by hanging than non-Indigenous youth (OR = 9.67, 95% CI: 6.55-14.26).

Among females, the odds of using hanging did not increase over time (OR = 1.00, 95% CI: 0.96–1.04; Table 3). Females’ use of hanging differed by age (*p* = .009), but the effect was nuanced. Compared to 10–14-year-old females, there was no difference in the odds of using hanging among 15–19-year-olds (OR = 0.58, 95% CI: 0.28–1.20) but the eldest group (20–24-years) had less than half the odds of using hanging (OR = 0.41, 95% CI: 0.20–0.85). There was some evidence that 20–24-year-old females were less likely to have used hanging than 15–19-year-olds (*p* = .023). Indigenous females had 6.5 times greater odds of dying by hanging than non-Indigenous females (OR = 6.54, 95% CI: 3.56-12.03).

Among males, in contrast, the odds of hanging increased over time (OR = 1.05, 95% CI: 1.02–1.07; Table 3). Furthermore, among males, use of hanging differed by age (*p* = 0.021). Compared to 10–14-year–old males, there was no difference in the odds of using hanging among either 15–19-year-olds (OR = 0.96, 95% CI: 0.55–1.66) or 20–24-year-olds (OR = 0.75, 95% CI: 0.44–1.29). However, 20–24-year-old males were less likely to have used hanging than 15–19-year-olds (*p* = .007). Indigenous males had 12 times greater odds of dying by hanging than non-indigenous males (OR = 12.14, 95% CI: 7.30-20.21).

#### Poisoning

Overall, as shown in Table 3, the odds of using drug-poisoning did not change over time among youth (*p* = .91). Compared with females, males had lower odds of using poisoning (OR = 0.29, 95% CI: 0.21–0.39). Compared to 10–14-year-olds, neither 15–19-year olds nor 20–24-year-old youth differed in their odds of using poisoning (*p* > .05), and Indigenous youth had lower odds of using poisoning than non-Indigenous youth (OR = 0.18, 95% CI: 0.72–0.43).

Among females, there was no evidence of change in the odds of using poisoning over time (OR = 1.01, 95% CI: 0.94–1.08; Table 3). There was evidence that females’ use of poisoning differed by age (*p* = .022). Compared to 10–14-year-old females, the odds of poisoning did not differ among 15–19-year-olds (OR = 1.85, 95% CI: 0.42–8.04) or 20–24-year-olds (OR = 3.30; 95% CI: 0.78–13.97). However, some evidence showed that 15–19-year-old females were less likely to have used poisoning than 20–24-year-olds (*p* = .017). Indigenous females had lower odds of dying by poisoning compared with other females (OR = 0.25, 95% CI: 0.09–0.69; Table 3).

Table 3 shows that, among males, there was no evidence that the odds of using poisoning changed over time or differed by age (Table 3). However, the odds of using poisoning were lower among Indigenous males compared with other males (OR = 0.08, 95% CI: 0.01–0.59; Table 3).

## Discussion

### Key findings

This study identified that whilst overall youth suicide rates did not increase significantly in Australia between 2004 and 2014, rates among females did, and this was consistently the case across all age groups and regardless of Aboriginal/Torres Strait Islander background. Rates were consistently higher among Aboriginal/Torres Strait Islander young people, among males, and older age groups (20–24-year-olds).

Throughout the study period, hanging was the most commonly used suicide method, and females and males were equally likely to have used hanging. Overall the odds of using hanging increased significantly over the period, although this was not the case among females.

### Interpretation

In contrast to earlier reports [11], this study found that overall rates of youth suicide did not increase significantly in Australia between 2004 and 2014. However, broadly stable rates among youth masked significant increases in young females. This finding adds to a growing body of research concerning the epidemiology of suicide among young females, which demonstrates increasing rates across OECD countries [4–8]. Importantly, the current study extended these findings to Australian females as young as 10 years old. Significant rate increases among younger females (10–14-year-olds) in this study are consistent with findings from the US and Canada, suggesting that an increasing number of early adolescent females are dying by suicide [6, 14]. This trend is concerning, given our findings that 10–14-year-old females showed greater odds of dying by hanging; a highly lethal method [40] that is difficult to restrict in community settings [41]. As such, these findings hold implications for prevention efforts.

Given crude rates in 10–14-year-olds were higher among females than males over several years, the gap in suicide rates typically observed between the sexes was not as evident in this younger age group. Rather, the rate ratio between the sexes appeared to widen with increasing age. This may be understood in light of earlier findings regarding sex and age differences in lethality of suicide attempts, which has shown that lethality tends to be lower among females than males for all methods [42], and elevated among younger age groups overall [43]. Therefore, sex differences in attempt lethality might depend on age. Future research linking sex-based trends in suicides with attempt data might thus elucidate whether the transition to adolescence marks the onset and peak lethality of suicide-related behavior in young females.

Contrary to expectations, no variable studied accounted for increased rates among females. Research highlights recent growth in the prevalence of modifiable risk factors that may explain an overall rise in young females’ vulnerability to suicide across groups, particularly when co-occurring within individuals [44]. These include concurrent increases in rates of depression and self-harm [30], alcohol misuse and related harms [45], and declines in vocational participation [46]. As this study focused on epidemiological risk factors coded in the NCIS, examining clinical risks and their cumulative effects was beyond our scope, but ought to be prioritized in future research to inform targeted interventions.

An unexpected finding was that females’ use of hanging showed no change over time, despite increasing among males and youth overall. This contrasts with prior studies that have consistently reported increases in the use of hanging among young females [19, 32, 33]. However, such studies did not report on younger-age females separately to older groups [32], and examined longer time-periods resulting in a larger number of cases [32, 33]. Our finding of no change in females’ use of hanging may be attributable to small annual counts over a constrained study period.

A novel finding was the emergence of distinct profiles of females who died by specific methods. Use of highly lethal methods (hanging) was more likely among 10–14-year-old and Aboriginal/Torres Strait Islander females, while methods less likely to be fatal (drug-poisoning) were more likely to have been used by 20–24-year-olds and non-Indigenous females by comparison. This is important as young people who self-poison are more likely to present to services and survive an index attempt [42, ﻿47], and are more likely to later die by drug-poisoning than other methods [47]. Taken together, opportunities to intervene following an index attempt may be greater among young adult and non-Indigenous females presenting to services following drug-poisoning. In such cases, the risk of dying by suicide following health service contact for an index attempt ought to be carefully evaluated and followed up by health professionals.

Groups demonstrating relatively higher odds of using hanging indicate that suicide attempts are more likely to be fatal in pre- and early-adolescent compared with young adult females, late adolescent compared with young adult males, and Indigenous young people of both sexes compared with their non-Indigenous peers. This might be particularly salient for younger Indigenous females, in whom suicide rates are both high and rising. The higher risk of cluster suicides among Indigenous youth [11] make this broad sub-group of young females a clear public health priority. Supporting local Indigenous communities to improve the social and emotional wellbeing of young people is crucial [48].

### Strengths and limitations

Key strengths of this study included comprehensive sampling of suicides among youth in Australia, including probable suicides, over an 11-year period. Additionally, this study reported on suicides among individuals as young as 10-years-old, and used standardized data thereby minimizing bias in reported estimates. Use of stratified mid-year population estimates facilitated standardization of population-stratified rates, controlling for annual variations in population distributions. Finally, this study employed statistical methods appropriate for empirically testing time trends for count data (negative binomial regression [49]), while multinomial logistic regressions examined trends in the use of specific methods.

Several limitations need to be acknowledged. Our model parameters assume a linear relationship between suicide prevalence and time whereas, in fact, suicide rates in both males and females fluctuated over time (Fig. 1). Therefore, whilst we can be confident that suicide rates increased over this time period in females, but not males, the observed magnitude of this increase may be different to that predicted by our model, highlighting the challenges in using linear prediction models to estimate changes in suicide rates. Relatively small annual counts may also have contributed to under-powered analyses in some instances, particularly among females. In particular, analyses relating to temporal changes in suicide rates for females between the ages of 10-14 years may have been under-powered given the model parameter estimates observed [50]. Therefore, whilst consistent with findings from other jurisdictions [6, 14], results for this group should be considered illuminative. A longer study period may facilitate aggregating counts over several years and improve statistical power for testing interaction effects.

The variables under study comprised a small proportion of established risk factors for youth suicide. Therefore, the scope of this study did not address potential contributions of clinical risk factors, including mental disorders and self-harm history. This, in turn, may have contributed to the discrepancy between our model parameters and the magnitude of change in suicide rates observed for both males and females over this time period. However, we restricted data to that reliably recorded by the NCIS to clarify the magnitude of rates and the contribution of key epidemiological risk factors. Additionally, by limiting our models to those factors that have been implicated in previous work as underlying the increase in suicide rates in females, we ensured our models were protected from over-fitting.

A final limitation relates to data coding. We included cases coded with ‘undetermined intent’ to capture probable suicides, as is conventionally reported in national statistics [50]. These cases were included in order to minimize previously reported underestimations, as coronial determination of intent is influenced by multiple factors including legal definitions and jurisdictional processes [37], as well as social and cultural sensitivities [51]. Prior to Australian data coding reforms in 2007, deaths that did not clearly meet criteria for a ruling of suicide, coded ‘accidental’, were later found to result in underestimations [37]. Additionally, data for 2013 and 2014 were incomplete at the time of writing [15] as data for equivocal deaths remain open for several years throughout the revisions process. Therefore, suicide counts for study years prior to 2007, and for 2013–2014, may be conservative. Variations in data collection are also reflected in incomplete data for Indigenous origin, which may be unavailable or not reliably reported [51]. We combined cases with Indigenous origin coded ‘unlikely to be known’ and ‘non-Indigenous’ into a single category in order to guard against inflating Indigenous suicide estimates, while retaining the total sample for analysis. However, this likely resulted in an underestimation of the true number of Indigenous suicide deaths, which could not be verified using the available coded data. Relatedly, the absolute number of suicides among Indigenous young people was low (although the relative risk was high), meaning that our estimates of risk for this group may be measured imprecisely.

### Implications for public health policy

This study highlights the importance of broadening current conceptualizations of youth suicide within public policy from that of a male problem to one increasingly involving young females. National policy on suicide prevention benefits from a greater understanding of recent time trends in young female suicides stratified by age and Indigenous background, which informs the need to target prevention efforts from a younger age and across both Indigenous and non-Indigenous groups. Current national approaches to suicide prevention in Australia may have overlooked preventative opportunities among young females, given females are more likely to seek help than males [30, 52].

A substantial body of research has shown that young females more often seek help for suicide-related behavior compared to males, including professional and non-professional sources of help [53]. Preliminary research within Victorian emergency departments has also shown that, among 12–24-year-olds, females make up the greater proportion of presentations for suicide-related behavior, and over half are sent home without a mental health assessment or referral [Donaldson A, Hetrick S, Redlich N, Spittal MJ, Robinson J: Youth Emergency Department Presentations for Self-Harm: A Retrospective File Audit Study, in preparation. Unpublished], despite heightened risk of suicide within 30 days post-discharge [54]. As such, females’ greater propensity for help-seeking, which can facilitate access to effective treatments [53], presents an important target for service reform. Such findings underscore a clear imperative for policy-makers to advocate for a more coordinated response to suicide-related behavior in young females, and to resource services accordingly.

At a population-level, restricting access to lethal means has proven to be an important and largely effective universal prevention strategy [55]. Although research highlights the potential for means restriction to reduce the population-level burden of suicide, difficulties with restricting hanging in community settings [41] necessitates the use of multiple evidence-based prevention strategies that target young people. Access to lethal means is a well-documented environmental precipitant that increases the risk of a fatal suicide attempt among young people [24], and hanging is both highly accessible and lethal. Combining alternative, evidence-based prevention strategies might include a combination of school-based awareness programs, gatekeeper training and screening, and cognitive behavioral and dialectical behavior therapies [56–59]. Such programs ought to be made available to younger age groups in recognition of the growing evidence that suicides are increasingly occurring among early adolescent females.

## Conclusions

In summary, this study expands current knowledge regarding the epidemiology of young female suicide in Australia, and reflects a broader trend of increasing young female suicide observed across the OECD. Stable rates among youth overall masked increases among young females between 2004 and 2014, highlighting the need for prevention strategies to address the rise in young female suicide, and, in particular, among Indigenous females in whom rates are considerably higher. While the majority of young females who died by suicide used highly lethal methods (hanging), this alone did not explain the rate increase. Rather, concurrent trends in rates of self-harm, depression and hospitalization for self-harm present a picture of morbidity and mortality that is complex, and indicate that vulnerability to suicide in young females is conferred by multiple risk factors which may exert a cumulative effect. However, the solution may be relatively straightforward. Young females engage in more help-seeking behavior, and more often speak about their difficulties with peers and professionals compared with young males. Therefore, opportunities exist to provide more targeted, responsive and effective support for young females, when and where they seek help.

## Data Availability

The suicide count data that support the findings of this study are available from the NCIS but restrictions apply to the availability of these data, which were used under license for the current study, and so are not publicly available. The mid-year estimated resident population datasets analyzed during the current study are available in the Australian Bureau of Statistics repository, http://stat.data.abs.gov.au/.

## Abbreviations

CI: Confidence interval
CMR: Crude mortality rate
ICD-10: International Statistical Classification of Diseases and Related Health Problems, 10th Revision
IRR: Incident Rate Ratio
NCIS: National Coronial Information System
OECD: Organization for Economic Co-operation and Development
OR: Odds Ratio
PY: Person-years
TSI: Torres Strait Islander
UK: United Kingdom
US: United States of America
